# Semi-automated protocol to quantify and characterize fluorescent three-dimensional vascular images

**DOI:** 10.1371/journal.pone.0289109

**Published:** 2024-05-16

**Authors:** Danny F. Xie, Christian Crouzet, Krystal LoPresti, Yuke Wang, Christopher Robinson, William Jones, Fjolla Muqolli, Chuo Fang, David H. Cribbs, Mark Fisher, Bernard Choi

**Affiliations:** 1 Beckman Laser Institute and Medical Clinic, University of California-Irvine, Irvine, CA, United States of America; 2 Department of Biomedical Engineering, University of California-Irvine, Irvine, CA, United States of America; 3 Department of Neurology, University of California-Irvine, Irvine, CA, United States of America; 4 Institute for Memory Impairments and Neurological Disorders, University of California-Irvine, Irvine, CA, United States of America; 5 Department of Pathology & Laboratory Medicine, University of California-Irvine, Irvine, CA, United States of America; University of Houston, UNITED STATES

## Abstract

The microvasculature facilitates gas exchange, provides nutrients to cells, and regulates blood flow in response to stimuli. Vascular abnormalities are an indicator of pathology for various conditions, such as compromised vessel integrity in small vessel disease and angiogenesis in tumors. Traditional immunohistochemistry enables the visualization of tissue cross-sections containing exogenously labeled vasculature. Although this approach can be utilized to quantify vascular changes within small fields of view, it is not a practical way to study the vasculature on the scale of whole organs. Three-dimensional (3D) imaging presents a more appropriate method to visualize the vascular architecture in tissue. Here we describe the complete protocol that we use to characterize the vasculature of different organs in mice encompassing the methods to fluorescently label vessels, optically clear tissue, collect 3D vascular images, and quantify these vascular images with a semi-automated approach. To validate the automated segmentation of vascular images, one user manually segmented one hundred random regions of interest across different vascular images. The automated segmentation results had an average sensitivity of 83±11% and an average specificity of 91±6% when compared to manual segmentation. Applying this procedure of image analysis presents a method to reliably quantify and characterize vascular networks in a timely fashion. This procedure is also applicable to other methods of tissue clearing and vascular labels that generate 3D images of microvasculature.

## Introduction

The microvasculature facilitates gas exchange, provides nutrients to cells, and regulates blood flow in response to stimuli [1]. Thus, it plays a fundamental role in the survival and health of tissues and organs. Vascular abnormalities indicate pathology for various conditions, such as compromised vessel integrity in cerebral microvascular disease and angiogenesis in tumors [2–4]. In addition, impaired blood flow to the brain is associated with neurodegenerative disorders such as Alzheimer’s disease [5, 6]. Traditional immunohistochemistry enables the visualization of tissue cross-sections containing exogenously labeled vasculature. Although this approach quantifies vascular changes within small fields of view, more practical ways exist to study the vasculature on the scale of whole organs. Furthermore, traditional immunohistochemistry requires tissue sectioning into thin (6–40 μm) sections, which effectively limits visualization of features to planar views and thus impedes facile 3D visualization of vascular architecture.

Volumetric imaging of tissue samples is vital to studying the microvasculature in its native state. The main limitation of 3D imaging of tissue samples is optical scattering resulting from the microscopic variations of refractive index occurring in most biological tissues. Organic and aqueous solvents reduce tissue turbidity by achieving refractive index matching [7, 8]. CLARITY is a popular tissue clearing method involving embedding of the sample into a hydrogel and using an electric current to remove lipids from the sample [9]. Other tissue clearing procedures have advantages and disadvantages associated with clearing time, the extent of clearing, and preservation of original tissue characteristics (e.g. size, endogenous fluorescence).

Quantitative analysis of microvasculature is essential to understand how structural variations in the microvascular network may change for different pathological states [10]. The feasibility of such analysis depends on accurate, ideally automated segmentation methods for isolating the microvasculature from the background due to the hundreds of gigabytes of data generated with high-resolution 3D imaging. Various groups have developed automated algorithms to perform quantitative characterization of vascular images [11, 12]. However, each method varies in complexity, processing time, and computational requirements.

We currently use iDISCO (immunolabeling-enabled three-dimensional imaging of solvent-cleared organs) as our primary tissue clearing method [7, 13]. Briefly, iDISCO consists of methanol dehydration, lipid removal with dichloromethane, and refractive index matching with dibenzyl ether. iDISCO is a fast, simple-to-implement clearing method that enables deep-tissue imaging. It is compatible with many exogenous labels often used for traditional immunohistochemistry. We previously demonstrated the effectiveness of iDISCO in combination with lectin-DyLight-649 for 3D visualization of vasculature in a mouse brain [13]. We also used Prussian blue labeling of hemosiderin [14–16], a by-product of cerebral microhemorrhages, with iDISCO-cleared brains and lectin-DyLight-649 labeling.

**Fig 1:**
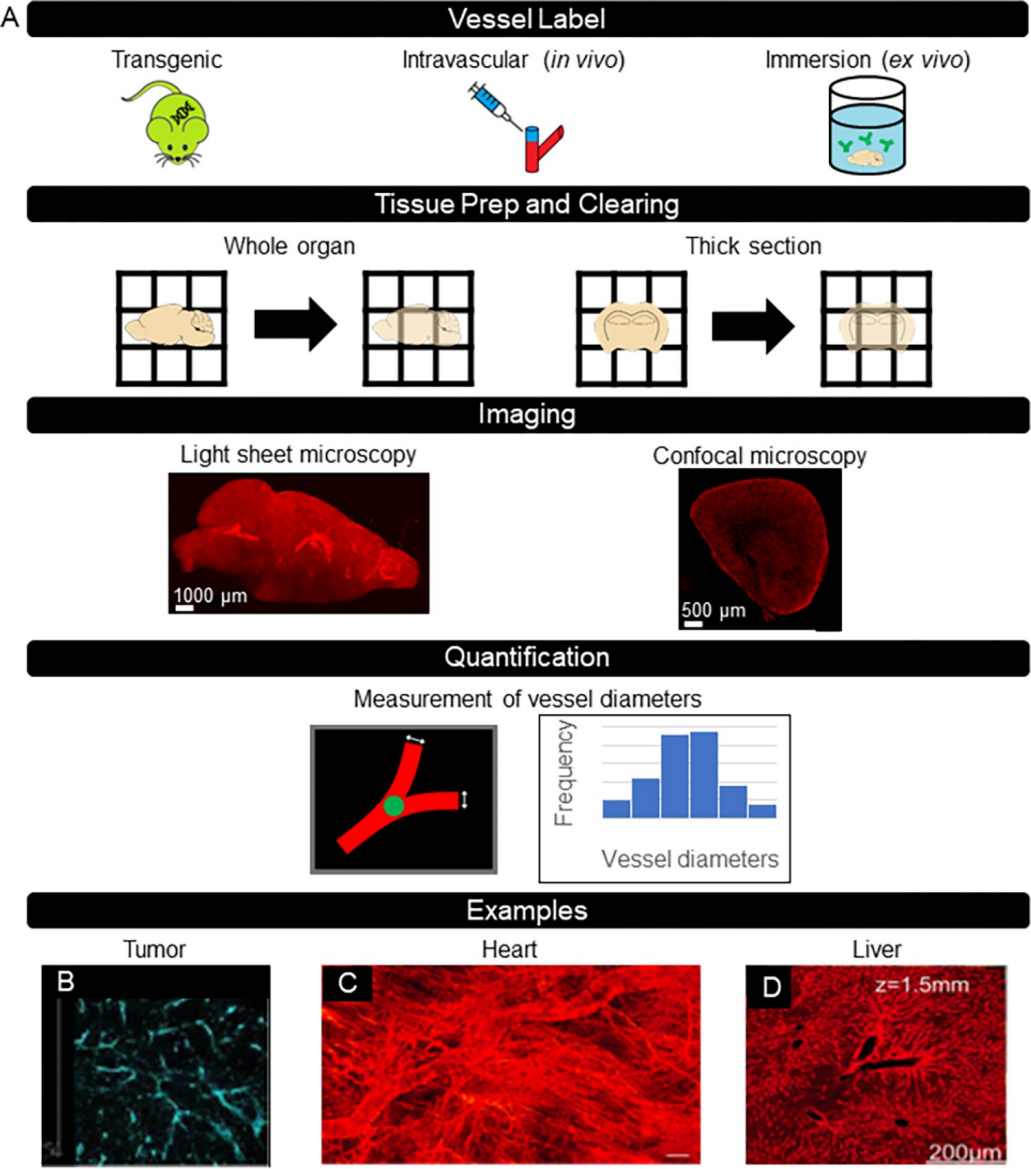
Overview of protocol for vascular visualization and quantification with various examples published in literature. A) Illustrations of the critical steps and workflow for vascular visualization in cleared tissue. B-D) Examples of vascular visualization in literature. B) Visualization of CD31-labeled mouse tumor vasculature with seeDB [30]. C) Visualization of DiI-labeled mouse heart vasculature with FocusClear [11]. D) Visualization of liver vasculature from *Tie2-Cre* mouse model with PEGASOS [33].

In this paper, we first review different methods for vascular labeling in conjunction with optical clearing that other groups have published. Next, we describe our complete protocol for imaging the vasculature in different organs in mice (Fig 1A). Specifically, we report on sample collection and perfusion of lectin-DyLight-649 followed by adding additional labels as desired and optical clearing. We then describe our procedure to obtain 3D confocal microscopy images. Finally, we describe our semi-automated approach to process the resulting images and quantify the vascular architecture in three dimensions. The proposed methodology consists of simple procedures that require minimal computational resources, which enables other researchers to produce accurate three-dimensional vascular images.

### Vascular imaging with various vessel labels and optical clearing techniques

Reviewing the literature on 3D vascular imaging of cleared tissue will show the large variability in methodology across different experiments (see Table 1 for specific references). For example, blood vessels can be visualized with transgenic animals expressing a fluorescent protein in endothelial cells, labeled *in vivo* with intravascular dyes, or labeled *ex vivo* with immersion-based antibody labeling. In addition, tissue preparation can vary from thick tissue sections and whole organs to whole-mount specimens, depending on the purpose of an experiment. Finally, clearing procedures can be grouped into aqueous solvents [17–22], organic solvents [12, 23–25], and hydrogel-based [26–29].

**Table 1:** Summary of different approaches to visualizing 3D microvasculature in optically cleared tissues.

Clearing agent(s)	Organ(s)	Vascular label	Quantification Method/Tool	Quantified Metrics
iDISCO [7]	brain, peripheral nervous system, kidney, muscle, heart, whisker pad, entire embryo	PeCAM	N/A	N/A
iDISCO [12]	brain	CD31, podocalyxin, collagen IV, smooth muscle actin, transgelin, von Willebrand factor	ClearMap 2.0, TubeMap	vascular density, vascular organization, vascular remodeling
MACS [17]	brain, heart, lung, spleen, femur, kidney, spinal cord, embryo, entire body	DiI, tetramethylrhodamine-conjugated dextran	Custom algorithm via Python, Imaris: surface function	glomerulus number and glomerulus volume; general metrics (imaging depth, size change, fluorescence intensity)
Sca*l*eS [18]	brain	Texas Red lectin	custom C++, commercial Volocity, commercial Igor Pro	microglia and Aβ plaques with custom C++, distance between microglia and Aβ plaques
CUBIC, BABB [19]	heart	FITC-lectin, 649-lectin, CD31	N/A	N/A
Sca*l*eS [20]	pancreas	lectin-DyLight	N/A	N/A
FocusClear [21]	small intestine	DiI	N/A	N/A
FocusClear, ScaleSQ, RIMS, sRIMS [22]	brain	Lectin-Dylight649	MATLAB	optical properties, vascular density
FDISCO [23]	brain, kidney	AlexaFluor647, CD31, lectin-DyLight-649	Imaris, ImageJ	general metrics (size change, imaging depth, fluorescence quantification)
3DISCO [24]	brain	FITC albumin-gelatin hydrogel	Imaris, ImageJ	vessel density, vessel diameter
vDISCO [25]	whole mouse	GFP, lectin	ImageJ, ClearMap	signal level, microglia distribution
CLARITY, TDE [26]	brain	lectin-FITC, gel-BSA-FITC, gel-BSA-TRITC	Markov random field-based algorithm, ImageJ, Amira 5.3 software	automatic segmentation, vessel diameter, vessel length
CLARITY [27]	brain	fluorescein-conjugated tomato lectin	Amira	area, volume, perimeter, and length of stained vessel
X-CLARITY [28]	placenta	DiI	Imaris, Image-Pro Premier	N/A
CLARITY [29]	retina	Griffonia lectin	Angiotool, ImageJ, Vaa3D, MATLAB, APP-2.0	network tracing, vessel percentage area, total number of junctions, junction density, total vessel length, average vessel length, total number of endpoints, mean lacunarity
PEGASOS [33]	whole mouse	αSMA, GS-IB4 isolectin dye, collagen IV	Imaris	vessel density
SeeDB, SeeDB2, 3DISCO, uDISCO, iDISCO, CUBIC, simplified CLARITY method, 75% v/v glycerol, Ce3D, FRUIT [34]	lymph nodes	CD31	Imaris: surface function, ImageJ	general metrics (imaging depth, size change, fluorescence intensity)
sodium dodecyl sulfate/sodium deoxycholate with Sca*I*eCUBIC-2 [35]	brain	RITC-Dex-GMA, Texas red lectin, CD31, αSMA	N/A	capillary diameters, signal-to-noise ratio
iDISCO with CUBIC [36]	ovary	endomucin	N/A	N/A
thiazone with PEG-400 [37]	dorsal skin	N/A (imaged blood flow via LSI)	N/A	blood flowblood flow via LSCI
iDISCO [38]	brain	Dye-conjugated secondary antibodies	ClearMap	Aβ deposits
FACT [39]	brain, spinal cord, heart, lung, adrenal gland, pancreas, liver, esophagus, duodenum, jejunum, ileum, muscle, bladder, ovary, uterus	CD31, autofluorescence	Imaris	segmentation
BABB [40]	heart	PECAM1	N/A	N/A
DBE, SCALE, CLARITY, CUBIC [41]	heart and embryo	GFP		
3DISCO [42]	brain	tomato lectin	N/A	N/A
CLARITY with ScaleA2 [43]	ovary, uterus, lung, liver	tdTomato	Ilastik, Imaris	segmentation, total vessel length, vessel mean diameter, vessel straightness, total number of branching points
ethyl-cinnamate [44]	liver, skin, lungs, heart, muscles, pancreas, brain, kidney	In-house developed NIR fluorescent dye (MHI148-PEI)	Leica LAS X	segmentation, volume of glomerulus
CLARITY, iDISCO [45]	brain	lectin-DyLight, lectin-FITC, anti-CD31	Imaris, segmentation	number of branches, total vessel length, total vessel volume, total vessel area, diameter of single segments per volume, distance between cells and nearest vessel
PEGASOS [46]	bone, teeth	tdTomato	Imaris	blood vessel volume
No clearing approach [47]	retina, heart	DiI	N/A	N/A
FRUIT 100 [48]	brain tumor	DiI	N/A	N/A
FocusClear [49]	brain	DiI	N/A	optical properties
FocusClear [50]	brain	DiI	MATLAB	functional vascular density

When selecting a clearing procedure, it is essential to consider its compatibility with the chosen vascular label. Endogenous fluorescence intensity is known to be reduced when using the iDISCO protocol; hence, an alternative vascular label would be preferred. Imaging of cleared samples is typically performed with confocal microscopy or light-sheet microscopy. Lastly, vascular-related metrics, such as vessel diameters and vessel density, are quantified with existing software such as FIJI and Imaris or custom-designed software such as ClearMap 2.0.

We have reviewed several optical clearing methods utilized to image vasculature. They are organized in Table 1 by clearing method, tissue type, vessel label, and quantification. Vascular images in cleared tissue from select publications are included in Fig 1B–1D. Lee et al. (2017) demonstrated the ability to use seeDB, a water-based clearing method that relies on fructose, to visualize CD31-labeled endothelial cells in whole mouse tumors (Fig 1B, 1B’) [30, 31]. Our group visualized vessels with an intravascular fluorescent dye and FocusClear (Fig 1C, 1C’) [11]. In addition, we quantified functional vascular density in different regions of cardiac tissue with a custom MATLAB algorithm [11, 32]. With a polyethylene glycol-associated solvent system (PEGASOS), researchers developed a clearing technique that preserved both hard and soft tissue structures (Fig 1D, 1D’) [33], allowing for vascular imaging throughout an entire specimen.

### Three-dimensional quantitative analysis of microvasculature imaged in thick tissue sections

Microvascular labeling is performed by injecting of a lectin conjugated to a fluorophore. In our work, we have focused on the DyLight-649 fluorophore. As the lectin travels through the circulatory system, it binds to glycoproteins adjacent to endothelial cells of the vascular wall. This binding allows for the labeling of the vascular network within every organ of the body.

**Fig 2:**
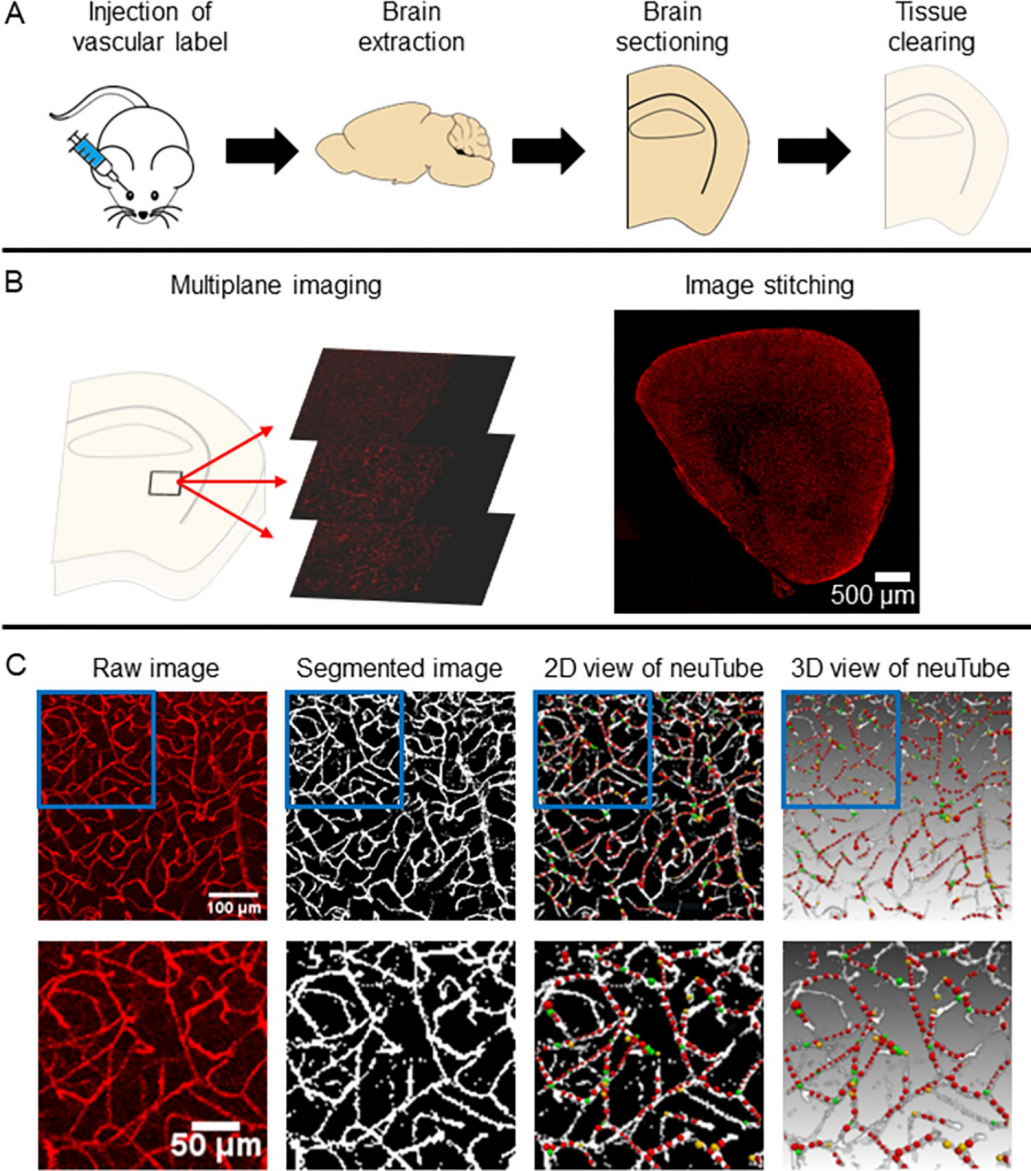
Workflow for 3D visualization and quantitation of microvasculature in thick tissue sections. A) Vasculature is labeled via retroorbital injection of lectin-DyLight-649. Brains are then sectioned and cleared. B) Depiction of multiplane imaging and image stitching to visualize an entire sample in 3D via confocal microscopy. C) From left to right: representative example of a raw image of the vasculature, segmented vasculature via MATLAB, 2D view of traced vasculature via neuTube, and 3D view of traced vasculature via neuTube.

An overview of our workflow is outlined below in Fig 2. First, we administer lectin-DyLight-649 via retroorbital injection. The lectin is allowed to circulate throughout the body before cardiac perfusion with saline, followed by formalin. Mouse brains are then extracted, bisected into hemispheres, and sectioned into thick (0.5–1.0mm) sections. Each section is then imaged using confocal microscopy to generate image stacks of the complete section throughout its entire thickness. Next, segmentation is performed by a custom MATLAB (MathWorks, Natick, MA) script. Finally, neuTube, an open-source neuron tracing software, is used to extract diameter measurements of the vessel structures [51].

## Materials and methods

The protocol described in this article is published on protocols.io (https://doi.org/10.17504/protocols.io.j8nlkk7m1l5r/v1) and is included as S1 File. All custom-written MATLAB code is available upon reasonable request made to the corresponding author.

### Animals

This work was in compliance with protocols approved by the University of California, Irvine Institutional Animal Care and Use Committee (approval number: AUP-19-084, AUP-19-032). Anesthesia is induced at 4% isoflurane within a chamber and maintained at 1.5% isoflurane. Euthanasia is performed via cardiac perfusion under 4% isoflurane anesthesia.

## Anticipated results

The procedures detailed herein present a simple method for producing optically cleared (> 1 mm thick) tissue samples with fluorescently labeled vasculature. Samples can then be imaged to generate 3D images of the microvasculature. The described segmentation method using custom MATLAB scripts and neuTube presents an automated method to convert the fluorescent images into binary images. The binary images can then be used to characterize microvasculature by quantifying vessel density and vessel sizes. The procedures here can be enhanced by using additional histology dyes or immunohistochemistry to label biomarkers or other structures of interest in tissue. In doing so, one can visualize and quantify the microvasculature surrounding these biomarkers to gain insight into their relationship.

## Validation

### Segmentation results

**Fig 3:**
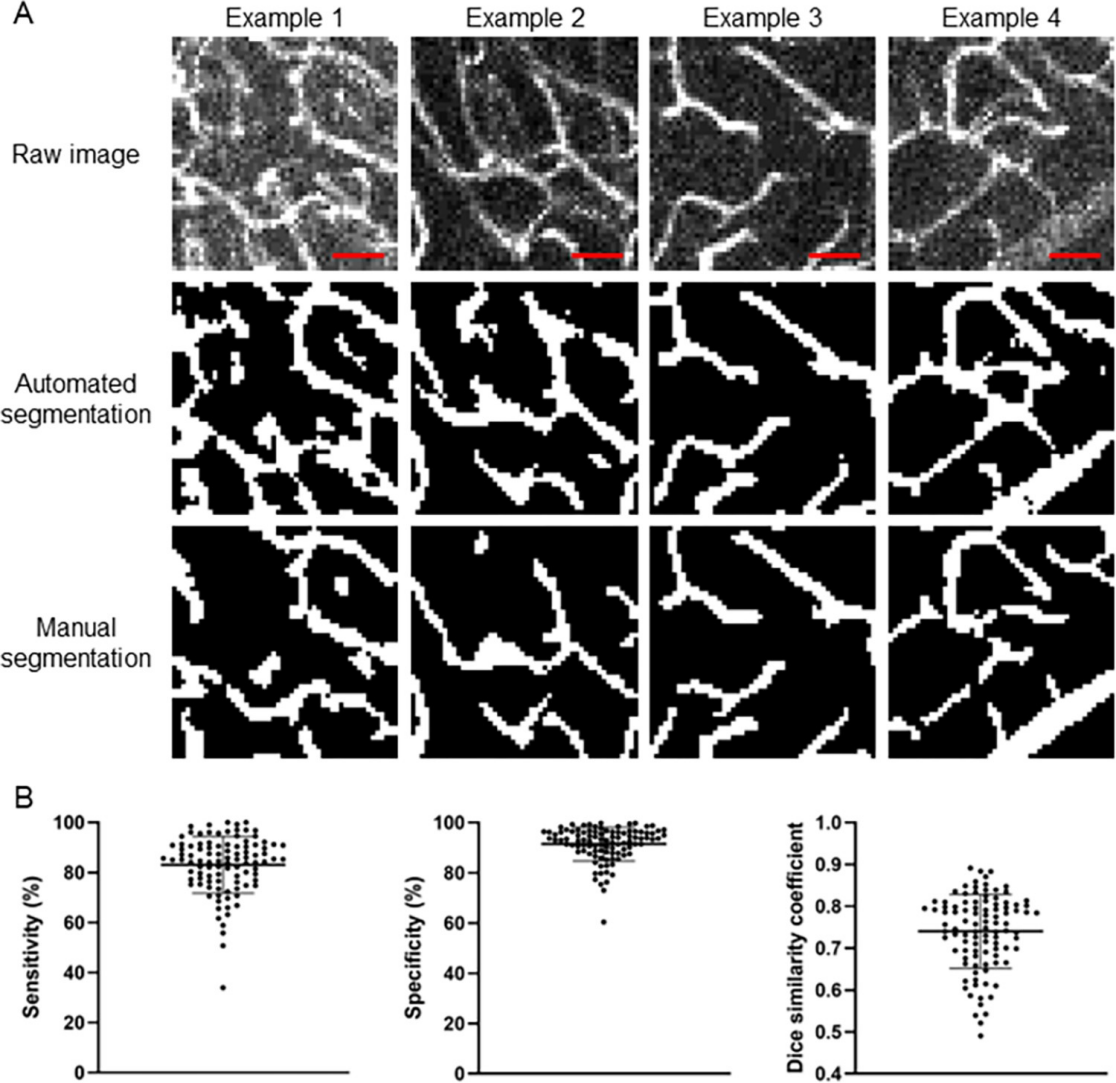
Representative examples of raw images, automated segmentation, and manual segmentation. A) Automated segmentation is performed using the iterative selection method as described in the step-by-step protocol (see S1 File). Manual segmentation is performed by manual tracing of vessels. Each example shown is a 50x50 pixel region from a different brain section. Scale bar is 25 μm. B) Comparison of vessel pixels identified via manual segmentation (ground truth) and automated segmentation. The sensitivity, specificity, and Dice similarity coefficient of all 100 ROIs are shown in the graph. The average sensitivity was 83±11%, the average specificity was 91±6%, and the average Dice similarity coefficient was 0.74±0.09.

To validate the accuracy of our automatically segmented images, manually segmented images were created to serve as a ground truth comparison. One author (DFX) used MATLAB to manually outline every pixel within an ROI of an image that the author determined to belong to a vessel. Each ROI (n = 100) was presented as a maximum intensity projection (MIP) image of a 50x50x5 voxel (113x113x38 μm) region. A MIP is used to present a 2D image that is more feasible for manual tracing. The corresponding 50x50x5 voxel region MIP from the automated segmented image was compared. Four representative examples of this are depicted in Fig 3. Across the 100 ROIs, the average sensitivity was 83±11%, and the average specificity was 91±6%. The Dice similarity coefficient between manually segmented and automated segmented images was calculated across all ROIs. This coefficient can measure similarity between two sets of Boolean data and ranges between 0 and 1, where 1 represents identical data, and 0 represents opposite data [52]. The average Dice similarity coefficient across 100 ROIs was 0.74±0.09.

### Diameter results with neuTube

**Fig 4:**
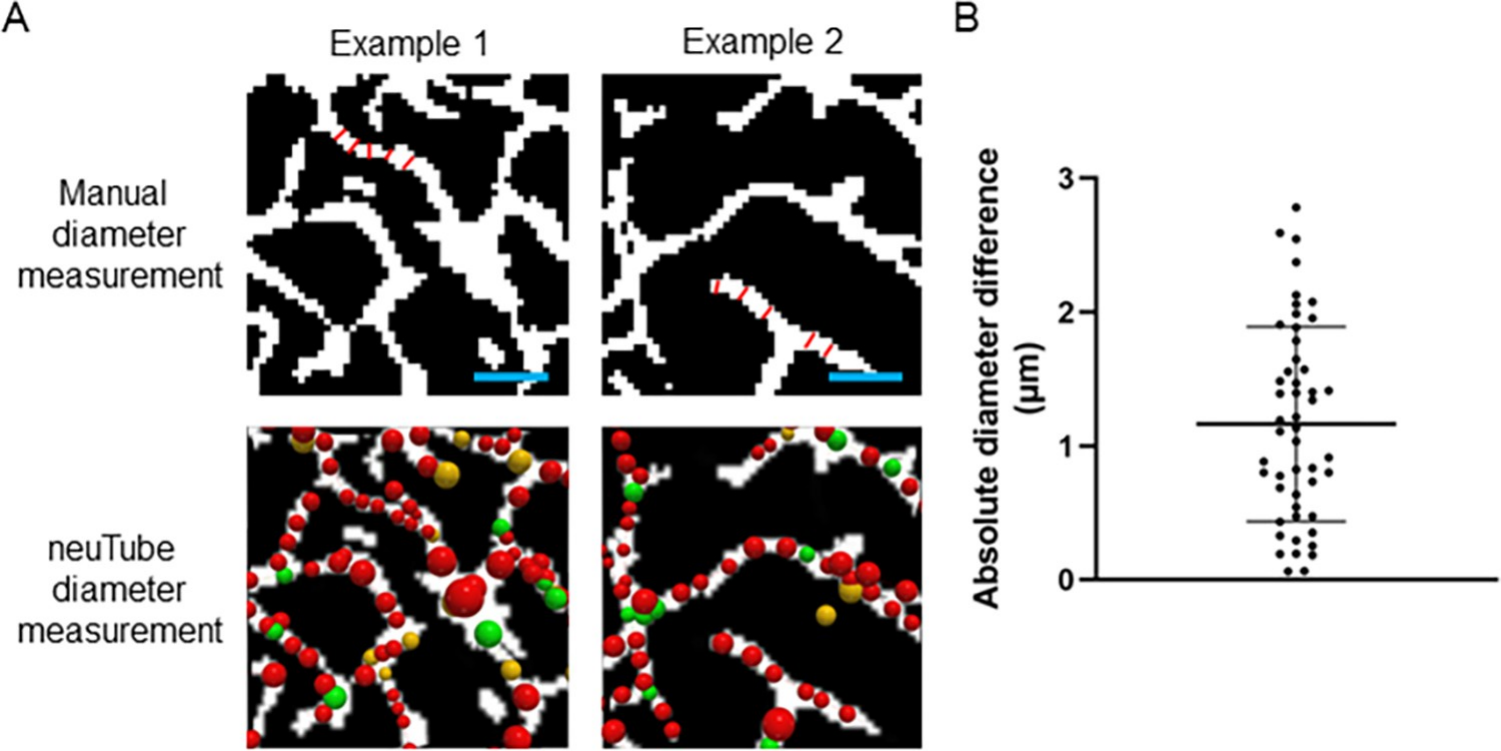
Two representative examples of manual diameter measurements and neuTube diameter measurements. A) Within a 50x50 pixel region, one vessel is chosen by the user for analysis. The manual diameter measurement is determined by drawing a line approximately perpendicular to the centerline of the vessel at 5 locations (shown with red lines in the top row of images). The manual diameter measurement for a given vessel is the average of these 5 measurements. The same vessel is identified within the automated neuTube output where each node (shown as a sphere in the bottom row of images) represents the diameter determined by neuTube at that location in the image. The neuTube diameter measurement for a given vessel is recorded as the average of five nodes along that vessel Scale bar is 25 μm. B) The absolute difference in diameter between manual measurement and neuTube measurement for 50 different vessels is shown. The mean difference was 1.16±0.73 μm.

Manually measured diameters of individual vessels were used as a ground truth comparison for neuTube approximated vessel diameters. Images from various brain sections across three different animals were used. One author (DFX) was presented with a randomly generated 50x50x5 voxel (113x113x38 μm) size region as a MIP. Within the image, the user was tasked with using MATLAB to estimate the diameter of a single vessel in the image at five different points along the vessel by drawing lines approximately perpendicular to the vessel’s centerline. In neuTube, five nodes along the corresponding vessel were selected. The average of the five manual diameter measurements via MATLAB and the five automated diameter measurements via neuTube were compared. Two representative examples of this are depicted in Fig 4A. The absolute difference between diameter measurements was 1.16±0.73 μm as shown in Fig 4B (n = 50 vessels of diameters ranging from 1.78 μm to 3.19 μm).

## Discussion

Due to the understood importance of cerebrovascular architecture to brain function, several groups have reported on similar methods to what we report here. Lugo-Hernandez et al. (2017) use an injected fluorescent-labeled hydrogel, the 3DISCO optical clearing approach, and light-sheet microscopy to image the vasculature, but vascular analysis is performed using a commercial software package (Imaris), which uses both user intervention and proprietary algorithms to achieve binary vascular maps [24]. Di Giovanna et al. (2018) employed a fluorescent gel perfusion approach to fill the vasculature, CLARITY-based optical clearing, and light-sheet microscopy; they describe the use of an automatic segmentation method based on a Markov random field, but enabling details of the algorithm are not provided [26]. Quintana et al. (2019) and Wälchli et al. (2021) used a vascular corrosion cast approach and micro-computerized tomography to visualize the whole-brain vasculature with exquisite detail, along with automated thresholding approaches (including the same iterative selection method we describe here); however, this approach requires access to a micro-CT system, and its compatibility with fluorescence labels requires further investigation [53, 54]. Kirst et al. (2020) report on the combination of immunolabeling, iDISCO-based optical clearing, and light-sheet microscopy to achieve whole-brain three-dimensional maps of the cerebrovasculature. They provide a sophisticated open-source software package to create binary maps of the vasculature, but a high-performance dedicated workstation is required to execute their computationally-intensive approach [12]. Hahn et al. (2021) used the same lectin-Dylight-649 fluorophore as we describe here, along with the FluoClearBABB optical clearing approach and light-sheet microscope; they describe an automated image analysis approach using a trained random forest classification scheme “ilastik” (Berg et al. 2019) [55, 56]. Takahashi et al. (2022) used the Cre/lox approach to induce Td tomato expression in endothelial cells, and combined this with CUBIC-based optical clearing and light-sheet microscopy to image the vasculature; they too used the ilastik software module for analysis [57]. Zhu et al. (2022) used different combinations of vascular labels and optical clearing approaches with light-sheet microscopy, but they also used Imaris for vascular image analysis [58].

With the work described here, we focused on describing the entire process of vessel labeling, brain extraction, and quantitative image analysis, on enabling other researchers to integrate and expand upon our protocol into their individualized workflow. We acknowledge that other vessel segmentation approaches may offer advantages to our approach described here, and the vetting of those approaches will be the subject of future work. Here, we emphasize the transparency of our methodology and ease of implementation of each step, with minimal computational resources, to achieve an approach that can rapidly yield accurate three-dimensional vascular maps. The vessel segmentation approach readily works with similarly-prepared whole brains imaged with light-sheet microscopy [13].

There are several potential applications for the methods described here. As angiogenesis occurs in organs, endothelial cells respond to local signals to adapt vessels to the surrounding environment [59]. Angiogenesis plays an essential role in tumor formation. In tumors, angiogenesis directly impacts tumor growth and metastasis. Overexpression of proangiogenic factors leads to uncontrolled vascular growth in tumors [60]. As a result, anti-angiogenic drugs are frequently used as a potential treatment option for cancer. Lectin-DyLight-649 combined with iDISCO presents a robust procedure for labeling the microvasculature in all body areas, including tumors. Changes in vessel density, tortuosity, and diameters can be quantified to evaluate the efficacy of novel anti-angiogenic treatments.

Ischemic strokes occur when there is a significant drop in cerebral blood flow due to occlusion in a cerebral artery. A major consequence of such an event is necrosis of neurons due to a deficient blood supply [61]. A method to visualize the microvascular network and the surrounding neurons, astrocytes, and glial cells can provide a detailed 3D snapshot of the brain in response to an ischemic stroke and monitor potential treatments over time.

In addition to visualizing cerebral microhemorrhages within the surrounding microvascular network and providing the improved capability to estimate the size range of these lesions, our approach can provide enhanced imaging for other disease entities. For example, a growing body of literature suggests a contribution of dysfunctional regulation of cerebral blood flow and various types of cognitive impairment [5]. The ability to visualize the cerebral microvasculature in three dimensions offers a new gateway, potentially leading to the identification of novel treatment targets for neurological disorders.

## Supporting information

S1 File Step-by-step protocol, also available on protocols.io. (https://www.protocols.io/private/653D11CD1E6A11ED93350A58A9FEAC02).
(PDF)
