# Supplementary material for: Semi-automated protocol to quantify and characterize fluorescent three-dimensional vascular images
Source: PLoS One. 2024 May 16;19(5):e0289109. doi: 10.1371/journal.pone.0289109 (PMC11098357; doi:10.1371/journal.pone.0289109)
Supplement: S1 File — (https://www.protocols.io/private/653D11CD1E6A11ED93350A58A9FEAC02). (PDF) [file pone.0289109.s001.pdf]

Semi-automated protocol to quantify and characterize fluorescent three-dimensional vascular images

Danny F Xie[1,2], Christian Crouzet[1,2], Krystal LoPresti[1,2], Yuke Wang[1,2], Christopher Robinson[1,2], William Jones[1], Fjolla Muqolli[1], Chuo Fang[3], David H. Cribbs[4], Mark Fisher[1,3,4,5], Bernard Choi[1,2]

[1]Beckman Laser Institute, University of California-Irvine;
[2]Department of Biomedical Engineering, University of California-Irvine;
[3]Department of Neurology, University of California-Irvine;
[4]Institute for Memory Impairments and Neurological Disorders, University of California-Irvine;
[5]Department of Pathology & Laboratory Medicine, University of California-Irvine

Danny F Xie

Protocol Info: Danny F Xie, Christian Crouzet, Krystal LoPresti, Yuke Wang, Christopher Robinson, William Jones, Fjolla Muqolli, Chuo Fang, David H. Cribbs, Mark Fisher, Bernard Choi . Semi-automated protocol to quantify and characterize fluorescent three-dimensional vascular images. **protocols.io** https://protocols.io/view/semi-automated-protocol-to-quantify-and-characteri-cffztjp6

Created: Aug 17, 2022

Last Modified: Mar 10, 2023

PROTOCOL integer ID: 68825

Keywords: vasculature, tissue clearing, image segmentation, image analysis, three-dimensional imaging

ABSTRACT

The microvasculature facilitates gas exchange, provides nutrients to cells, and regulates blood flow in response to stimuli. Vascular abnormalities are an indicator of pathology for various conditions, such as compromised vessel integrity in small vessel disease and angiogenesis in tumors. Traditional immunohistochemistry enables visualization of tissue cross-sections containing exogenously labeled vasculature. Although this approach can be utilized to quantify vascular changes within small fields-of-view, it is not a practical way to study the vasculature on the scale of whole organs. Three-dimensional (3D) imaging presents a more appropriate method to visualize the vascular architecture in tissue. Here we describe the complete protocol that we use to characterize the vasculature of different organs in mice encompassing the methods to fluorescently label vessels, optically clear tissue, collect 3D vascular images, and quantify these vascular images with a semi-automated approach. Applying this procedure of image analysis presents a method to reliably quantify and characterize vascular networks in a timely fashion. This procedure is also applicable to other methods of tissue clearing and vascular labels that generate 3D images of microvasculature.

MATERIALS

Reagents:

- Saline solution (Aspen Veterinary Resources, NDC No. 46066-807-50)
- 10% formalin solution (Sigma-Aldrich Product Code HT50-1-1)
- PBS with 0.02% sodium azide solution (Syringa Lab Supplies, Part No. 11001)
- Potassium ferrocyanide (Sigma: P3289-500G)
- Hydrochloric acid (Fisher: A144-212)
- Methanol (Fisher: A412S)

- Deionized water
- Dichloromethane (Sigma: 270997-100ML)
- Dibenzyl ether (Sigma: 108014-1KG)

Equipment:
- 2x 10 mL syringes (Sigma-Aldrich: Z683604)
- 1x 23G x ¾ x 12” butterfly needle (Vaculet, 26766)
- 3x hemostat clamps (Excelta: 37SE)
- 1x bone scissors (Fine Science Tools: 91604-09)
- 1x forceps (Fine Science Tools: 11000-12)
- 1x scissors (Excelta: 290)
- 1x angled scissors (Fine Science Tools: 15010-10)
- 1x spatula (Fine Science Tools: 10089-11)
- 1x syringe pump (Harvard Apparatus Model 11 Plus)
- 1x 20 mL glass scintillation vial (Grainger: 3LDT2)
- Aluminum foil
- Tape
- Liquid absorbent mats
- Isoflurane chamber (E-Z Systems: EZ-178)
- Nose cone (E-Z Systems: EZ-103A)
- Magnetic stir plate with stir bar (Corning PC 353 Stirrer)
- Orbital shaker (Scilogex: SK-D1807-E)
- Scale
- Weigh boats
- Pipette controller (Grainger: 49WF85)
- Serological pipette tips
- 1.5 mL opaque microcentrifuge tube (Argos Technologies: 06333-80)
- Microcentrifuge tube rack
- Pipettes
- 18-gauge needle
- Leica TCS SP8 microscope

Software:
- MATLAB (https://www.mathworks.com/)
- FIJI (https://imagej.net/Fiji)
- neuTube (https://www.neutracing.com/)

Reagent setup:
- *10% potassium ferrocyanide (PF) solution*

Dissolve the appropriate amount of PF in deionized water (DIW) (10 g of PF per 100 mL of DIW) to create a 10% PF solution. Use a magnetic stir bar and magnetic stir plate to dissolve potassium ferrocyanide.
- *20% hydrochloric acid (HCl) solution*

Dilute the appropriate amount of stock HCl solution in DIW (20 mL of stock HCl per 80 mL of DIW) to create 20% HCl solution. Perform this step under a fume hood.
- *Potassium ferrocyanide/hydrochloric acid working solution*

Mix 1 part 10% potassium ferrocyanide solution per 1 part 20% HCl solution

Cardiac perfusion and retrooribtal injection

45m

1 Cardiac perfusion

1.1 Begin by anesthetizing a mouse using an isoflurane chamber with 1.5 L/min of oxygen and 4.0% isoflurane.

1.2 Once the mouse is anesthetized, remove it from the chamber and place its snout in a nose cone at 1.5 L/min oxygen and 1.5% isoflurane.

1.3 Administer a solution of lectin-DyLight-649 (200 µL, 25% lectin-DyLight and 75% saline) via retroorbital injection.

CITATION

Prabhakar S, Lule S, da Hora CC, Breakefield XO, Cheah PS (2021). AAV9 transduction mediated by systemic delivery of vector via retro-orbital injection in newborn, neonatal and juvenile mice.. Experimental animals.

LINK

https://doi.org/10.1538/expanim.20-0186

1.4 Allow the solution to circulate for about 20 minutes before proceeding with the cardiac perfusion. 20m

1.5 It is recommended to perform the cardiac perfusion on a surgical tray or similar platform to contain the exsanguinated blood. Confirm that the mouse is at an appropriate plane of anesthesia using toe and/or tail pinches.

1.6 Next, open the chest cavity by performing a horizontal incision beneath the rib cage and a vertical incision along both sides of the chest.

.

CITATION

Gage GJ, Kipke DR, Shain W (2012). Whole animal perfusion fixation for rodents.. Journal of visualized experiments : JoVE.

LINK

https://doi.org/pii:3564.10.3791/3564

1.7 Use hemostats to assist with holding the chest open to access the heart.

1.8 Perform a small incision on the right atrium of the heart to allow blood to exit the body.

1.9 Insert a butterfly needle into the left ventricle of the heart. Use a syringe pump to perfuse 10 mL of saline into the heart at a rate of 2 mL/min. 5m

1.10 Perfuse 10 mL of formalin at a rate of 2 mL/min 5m

Brain extraction

10m

2 Brain extraction

2.1 Remove the head by cutting caudal of the skull with scissors (bone scissors or large scissors preferred).

2.2 Cut the scalp to create two folds and expose the cranium.

2.3 Using angled microscissors, gently cut upwards from the foramen magnum to approximately the location of bregma on the brain.

2.4 Using a set of scissors, perform a lateral cut directly rostral to the olfactory bulbs. This cut should split the remainder of the skull down the centerline beyond bregma.

2.5 Use fine tweezers or a spatula to pry open each hemisphere of the skull.

2.6 Gently separate the brain from the base of the skull with a spatula. Sever any nerves connecting the brain to the skull.

2.7 Place the brain in ~10 mL of 10% formalin to completely submerge the brain. Store away from light.

2.8 After 24h, store the brain in PBS with 0.02% sodium azide at 4°C until further tissue processing. Store away from light.

(Optional) Exogenous labeling of hemosiderin with Prussian...

1h 30m

3 The following procedures are based on 1-mm thick coronal sections of a bisected brain. Volumes and times may need to be adjusted for tissues of different sizes.

3.1 Prepare a working solution of 10% w/v of potassium ferrocyanide (10 g of potassium ferrocyanide per 100 mL of DIW).

3.2 Use a magnetic stir plate to mix the solution for at least 20 minutes.

3.3 Prepare a working solution of hydrochloric acid that is 20% of stock hydrochloric acid.

3.4 Mix the solutions of potassium ferrocyanide and hydrochloric acid in a 1:1 ratio. Approximately 5 mL of the mixed solution is used per sample. This solution should be prepared prior to each staining session.

3.5 Wash samples in 5 mL of DIW with shaking.

3.6 Submerge each sample into 5 mL of the working potassium ferrocyanide/hydrochloric acid solution for 1 hour

3.7 Perform a final DIW wash for 5 minutes.

3.8 Store the samples in PBS with 0.02% sodium azide at 4°C and away from light.

Tissue clearing

3h 30m

4 The following procedure is modified from the established iDISCO protocols. Wash durations were modified for 1-mm thick coronal sections of a bisected brain.

CITATION

Renier N, Wu Z, Simon DJ, Yang J, Ariel P, Tessier-Lavigne M (2014). iDISCO: a simple, rapid method to immunolabel large tissue samples for volume imaging.. Cell.

LINK

https://doi.org/10.1016/j.cell.2014.10.010

4.1 Perform a series of methanol washes (20%, 40%, 60%, 80%, 100%, and 100%, balance DIW) each for 20 minutes with shaking. Microcentrifuge tubes of 1.5 mL (or larger for larger samples) are recommended. Fill the tubes fully to minimize exposure to oxygen.

4.2 Incubate the samples in a solution of 66% dichloromethane and 33% methanol for one hour with shaking. The sample may be stored overnight in this situation if desired, without shaking.

4.3 Incubate the sample in dichloromethane twice for 15 minutes with shaking.

4.4 Store the samples in dibenzyl ether at 4°C until imaging.

Imaging with confocal microscopy

5 When imaging samples that have been cleared following the iDISCO protocol, it is recommended to image the sample while submerged in dibenzyl ether. There are various methods to safely house a sample with dibenzyl ether and protect the imaging objective. A straightforward method is to create an epoxy well on a cover glass to surround the sample and hold the dibenzyl ether.

5.1 To image the vasculature labeled with the lectin-DyLight-649, use an appropriate wavelength (633 nm can work) with an emission band of approximately 650-750 nm.

5.2 To visualize microhemorrhages labeled with Prussian blue, locate regions of Prussian blue positivity using a white light source.

5.3 Simultaneously collect a fluorescent image of the vasculature and a transmittance image to co-register the vascular fluorescence with cerebral microhemorrhages. This workflow along with representative images are depicted in **Fig 1**.

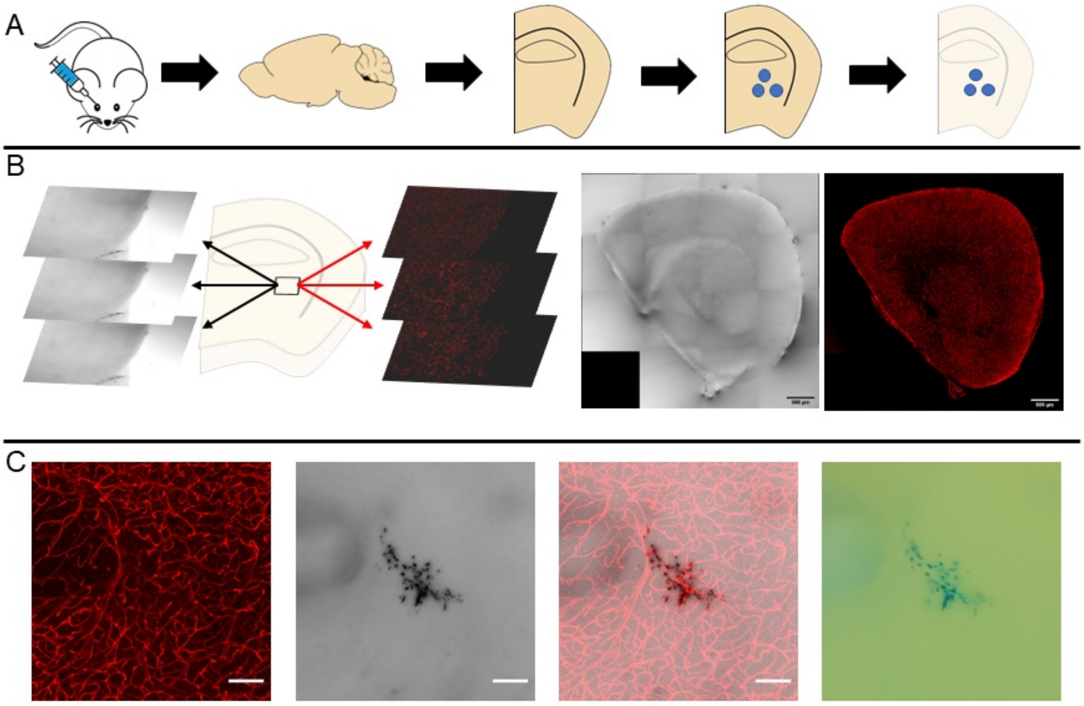

Fig 1. Workflow for 3D visualization of cerebral microvasculature cerebral microhemorrhage in thick tissue sections. A) Vasculature is labeled via retroorbital injection of lectin-DyLight-649. Brains are then sectioned, stained, and cleared. B) Depiction of z-stack imaging and tile stitching for a fluorescence channel and a transmission channel via confocal microscopy. C) From left to right: fluorescent image of microvascular network in a 70 µm thick tissue region, transmission image of cerebral microhemorrhage in the same tissue region, overlayed image of both imaging channels, eyepiece view of Prussian blue positive cerebral microhemorrhage. Scale bars are 100 µm.

5.4 Our presented results are primarily from brain samples, but the procedures can be easily translated to other organs. Light-sheet microscopy can be used as an alternative to confocal microscopy to rapidly generate 3D reconstructions of the vascular network with minimal photobleaching.

CITATION

Khouri K, Xie DF, Crouzet C, Bahani AW, Cribbs DH, Fisher MJ, Choi B (2021). Simple methodology to visualize whole-brain microvasculature in three dimensions.. Neurophotonics.

LINK

https://doi.org/10.1117/1.NPh.8.2.025004

Fig 2 shows representative images of different labeled and cleared organs imaged with confocal microscopy and light-sheet microscopy.

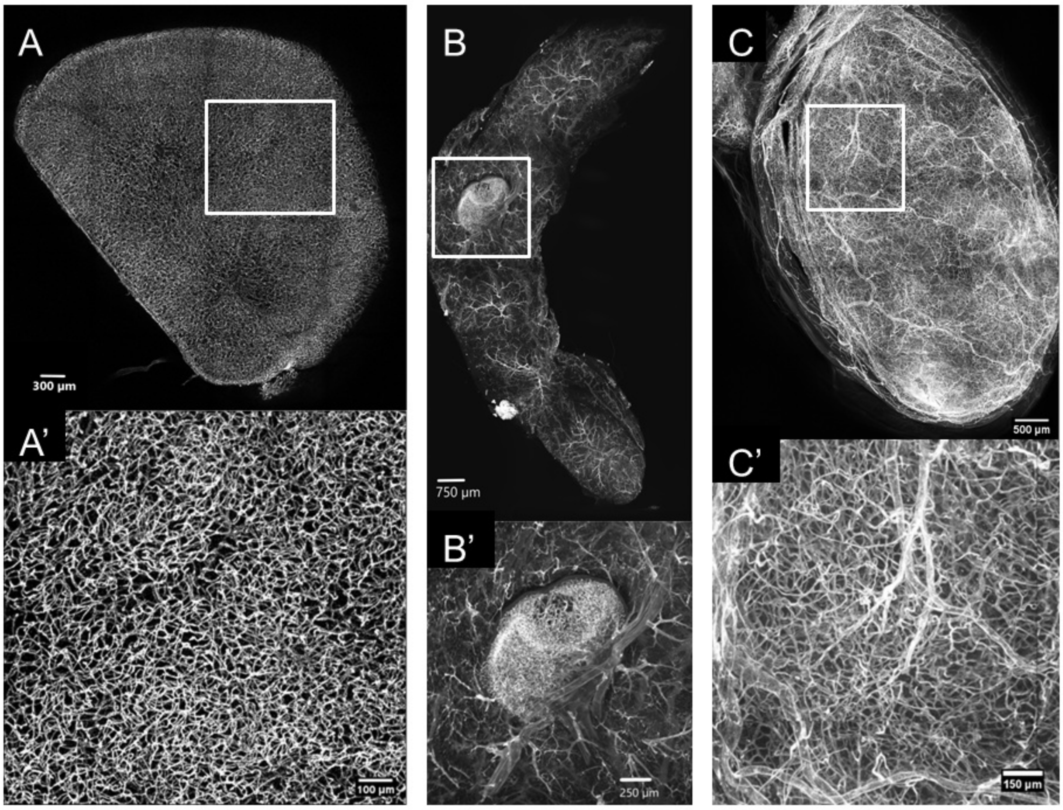

Fig 2. Lectin-DyLight-649 labeling and iDISCO optical clearing enable detailed visualization of mouse organ microvasculature. A) Confocal image of a 1-mm thick coronal hemisection of the brain. A') Magnified view of A. B) Light-sheet image of a mammary gland with a lymph node. B') Magnified view of B. C) Light-sheet image of a bladder. C') Magnified view of C.

Vascular segmentation

6 There are various segmentation methods to isolate the fluorescent vasculature from the image background. The iterative selection thresholding method was implemented to binarize the fluorescent images. This algorithm was selected due to its simplicity and objectivity and was performed using custom-written code in MATLAB.

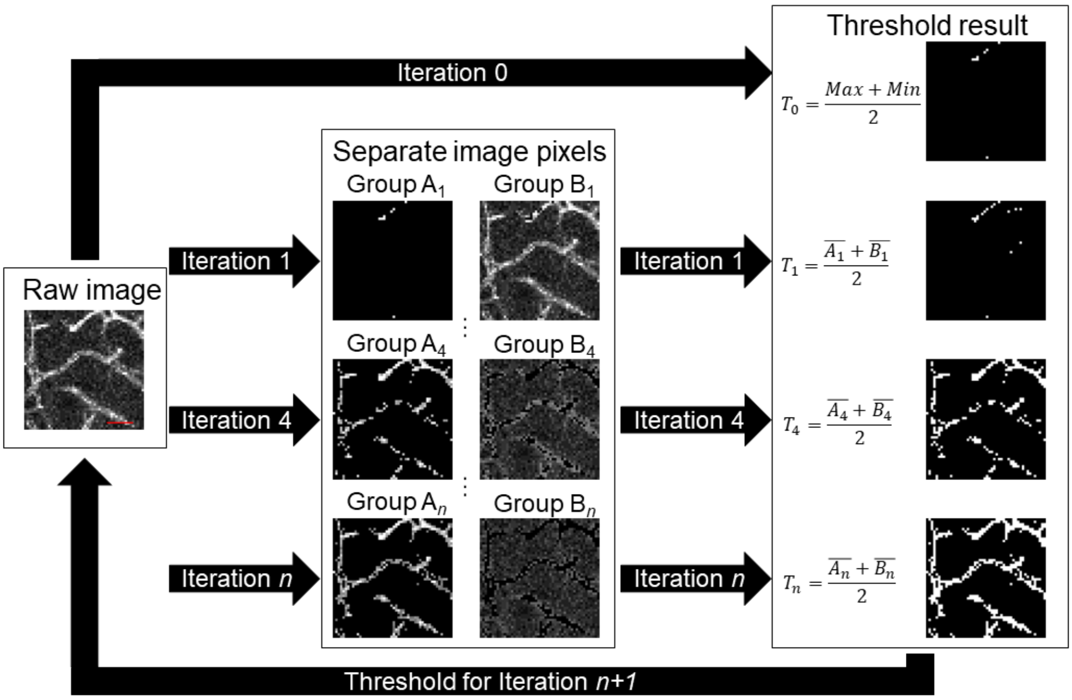

Fig 3. Schematic of iterative selection thresholding method for images of fluorescent vasculature.

6.1 Use a 3x3x1 median filter to remove noise within the image data. This step can be skipped if the noise in the image is sufficiently low.

6.2 The iterative selection threshold method is applied by selecting an initial threshold value, T_0. The initial value is the average value of the maximum intensity and the minimum intensity of all voxels of an image.

$$T_0 = \frac{Max+Min}{2}$$

All voxels within an image are separated into two groups: group A_n if a voxel has an intensity equal to or greater than the threshold value of the previous iteration, or group B_n if a voxel has an intensity below the threshold value of the previous iteration.

$$A_n \ge T_n, B_n < T_n$$

The threshold value of the subsequent iteration is calculated as the average value of the average intensity within groups A_n and B_n.

$$T_n = \frac{\bar{A}_n+\bar{B}_n}{2}$$

This threshold value is compared to the threshold value of the previous iteration. If the difference between the two values is less than 1, the procedure is complete, and the current threshold value is selected. If the difference between the two values is greater than or equal to 1, the procedure repeats for another iteration.

$$If~|T_n-T_{n-1}| < 1,~then~stop$$

6.3 Optional morphological operations can be applied to the resulting binarized image to adjust segmentation results further. The exact parameters for these steps will vary with image acquisition parameters (particularly resolution).

neuTube tracing to quantify vessel diameters

7 neuTube is an open-source neuron tracing software that can be applied to tracing tubular structures, such as vasculature.

CITATION

Feng L, Zhao T, Kim J (2015). neuTube 1.0: A New Design for Efficient Neuron Reconstruction Software Based on the SWC Format.. eNeuro.

LINK

https://doi.org/pii:ENEURO.0049-14.2014.10.1523/ENEURO.0049-14.2014

7.1 Automated tracing can be performed on binarized vasculature images in TIF file format. neuTube will output information in an SWC format where tubular structures are simplified into individual nodes with x, y, z coordinates, a radius, and node connectivity information. Each node will be a ‘parent’ to an adjacent ‘child’ node which provides the necessary information to understand how these nodes are connected in space.
